# Half-Yearly Report of the Progress of Medicine, from January to June, 1813

**Published:** 1813-07

**Authors:** 


### Medical and Physical Journal.

1 OF VOL. XXX.] JULY, 1813. [NO. 173.

*For the Medical and Physical Journal.*

### HALF-YEARLY REPORT *of the* PROGRESS *of* MEDICINE, *from* JANUARY *to* JUNE, 1813.

(No. VIII.)

"O beata Sanitas! te præsente, amænum

Ver floret gratiis, absque nemo beatus."

"Daughter of Pæan, queen of every joy,
HYGEIA! whose indulgent smile sustains
The various race luxuriant nature pours,
And on th' immortal essences bestows
Immortal youth; auspicious, O descend!"

THAT the legitimate professors of the medical art were solicitous only, and above all things, to prevent disease or to restore health, has been believed against the surly argument of the misanthrope, and the flippant sarcasm of the wit: recent events have, however, shaken that faith, but soon to be restored, it is hoped, to that confidence and trust on which the mind can repose with unapprehensive satisfaction.

The leading feature of our last REPORT was drawn from the effort then making by a vast majority of the medical faculty to meliorate professional education, to provide for society practitioners of higher qualification, and to place around the public health a barrier sufficiently substantial to repel the fool-hardiness of ignorance, and impenetrable to the impudence and cunning of an empiricism which had too long been suffered to undermine the springs of health and life. A narrowness of plan, and an apparent, but only apparent, selfishness, embarrassed and retarded, at the outset, this this great public measure. We spoke of it as we felt, and take some credit for having suggested an extension which will finally accomplish an object that must, in its results, benefit the empire, by substituting for the heterogeneous mass* now assuming the title and functions of medical practitioners, persons properly educated, of ascertained qualifications, respectable by the class of society from whence they will be taken, and by having passed through the series of essential gradations.

---

* Can this require explanation? The mass of the medical faculty of the British empire is, indeed, an heterogeneous compound. Physicians, doctors of the English universities, fellows of the Royal College, versed in all human science, learned, honorable, and approximating to the "corinthian capital of society," are "pushed from their seats" by doctors of no universities, unlearned men and even women, without science, and without honor. The Abernethys, Coopers, and Brookes's, have opposed to them self-created surgeons, raspers of shin-bones, advertising gonorrhœa cures, and men whose chirurgical knowledge has been acquired by carrying a box after the dresser at an hospital. The legitimate apothecary is circumvented by the druggist who was yesterday a grocer; by the chemist who hardly knows a crucible from a cauliflower. To the educated physician, to the regular surgeon, to the instructed apothecary, this is personally, perhaps, unimportant; but what is it to the public? pain, mutilation, death. London, that common sewer, teems with this surreptitious multitude. From Tower-hill, a doctor advertises that "all persons afflicted with any complaint *whatever*, may have an easy, speedy, and certain cure, without confinement," by applying to the said doctor. Mrs. ——— cures the king's evil radically, and, what is more, safely too. Another benevolent female, at Battle-bridge, continues, as usual, to cure consumptions; and still another at Islington, cures cancer. Even at the college-gate of Edinburgh lived, very lately, a female "*practitioner in medicine*" from England; and the druggists of a midland county at this moment are querulously complaining that a horrible attempt is making to take from them the privilege of prescribing for the sick. We are not among those who think this must be endured, but believe that laws may be framed to protect society against these depredators, not on money but on health. If it is the fiat of Providence that good and evil be mingled, and that in the moral as in the natural world the noxious plant shall grow beside the salutiferous herb,

> Terra salutiferas herbas, eademque; nocentes
> Nutrit, et urticæ proxima sæpe rosa est; the gardener may exercise his office; the poisonous may be extirpated, and the doubtful confined to their quarters.

Those

Those who have read the *Reports* on the progress of the medical art, which for some years have been published in this Journal, will readily appreciate the anxiety always there manifested for the improvement of, indeed, a collateral and auxiliary branch of that art, but, from its operation upon the whole system of medical science, of intrinsic importance. Under the comprehensive term MEDICAL JURISPRUDENCE, exists a master movement, which may, when duly organized, direct, control, inform, and animate, the whole machine.

The association formed by the apothecaries and surgeon-apothecaries of England and Wales, has a direct reference, in its views and operation, to a leading principle in a *system of medical police.* To improve in knowledge and advance in respectability this class of practitioners, surely involves a great public benefit: to prevent the idle, the dissolute, the ignorant, or the cunning empiric obtruding on society, under any anomalous appellation, with his balms, his balsams, and his cordials, must surely lead to public safety.

Acting, as we think, on this principle, our last *Report* left the *Committee* of the "COMMUNITY *of* ASSOCIATED APOTHECARIES *and* SURGEON-APOTHECARIES" adjusting and regulating their purposed application to the legislature. The opinion, advice, and assistance of this branch of the faculty had been collected from the provinces; and the deputies of numerous districts attended a general meeting of the Association in London, in the month of March. Consequent on this meeting, the purposed application, the preliminary steps for which had before been taken in the usual form of a Bill presented to the House of Commons, was determined to be vigorously pursued. But fortunately, perhaps, for the cause, which a spirit of public benevolence must countenance and cherish, this Bill, immature, hurried, imperfect, and defective in detail, though incontestibly sound in principle, was withdrawn before the second reading, to be revised and improved, with the avowed intention of again submitting it to parliament.

On some peculiarities of this Bill, and on the combination of causes that induced to its postponement, we hazard a few cursory observations.

It is evident that in framing this Bill the Committee must have had many jarring interests to reconcile, arising even in the body to which it particularly applied; much external opposition to conciliate or to conquer, arising out of public opinion, or out of private apprehension. Some thought it too extended; others esteemed it too limited. Some asserted it did nothing if it did not annihilate the druggist. Some contended for the apothecaries' right to demand fees. One district had this, another had that, local interest to satisfy. Pressed by the multitude of claims, one often in direct opposition to another, and desirous, probably, to attend to every demand, and to gratify every wish, the Committee seems to have lost sight of, or to have mixed too much with other views, the main spring and principle of their project. To improve the school education, *ab initio*, of the apothecary and surgeon-apothecary; to perfect the students of that class in the elements of science; to prevent unqualified persons entering this department of the profession, by the barrier of a strict examination; seem to have been, and should continue to be, the vital principle animating and directing every view and motive of this application to the legislature. But embarrassed, probably, by intricacy of interests, and operated on by the influences above stated, the Committee suffered clauses, very loosely connected with this great principle, to be inserted in the Bill. The incongruity and inequitable extent of these hastily admitted clauses, a knowledge of the public opinion respecting them, and an ascertainment of the opposition to which they gave rise, determined the Committee to postpone the prosecution of the measure at that time.

That the objections to this Bill, founded on the clauses above alluded to, so far as those clauses went, were legitimate, will hardly be denied; but how they gave rise to another very formidable portion of opposition, will not easily be proved, but by that opposition being withdrawn with the objectionable clauses. That the privileges and rights of the College of Physicians and the Court of Assistants of the College lege of Surgeons could be affected by this Bill in even its most objectionable form, we are not able to say; but we know that the public would have been largely, essentially, and permanently benefited by passing it into a law, even in its faulty state. Under its auspices and operation, a race of practitioners to whom the public health is principally entrusted, would have necessarily arisen, improved in every thing that is valuable. It would also have possessed the inestimable property of inducing progressive advancement towards perfection in an employment, the objects of which are of no less magnitude than the health and lives of the population.

That a measure of this public utility, (for who will say that improvement in medical knowledge, and an assurance that no person ignorant of the art could thereafter practise medicine, is not a measure of public utility?) should have an interest opposite to that of the Royal College of Physicians, and the Court of Assistants of the College of Surgeons, must be viewed as a serious misfortune. But how these bodies will justify their opposition to a measure demonstrably beneficial to society, upon the ground of its becoming, probably and eventually, injurious to their corporations, will be looked for with anxious curiosity. Our respect for these bodies will make us lament that the public should inquire if it be essential to the support of their preponderance, wealth, and influence, that the Apothecary and Surgeon-Apothecary, to whom the health of the community is entrusted in the proportion of ten to one, should be ignorant and depressed, debased by the intrusion of empiricism, and subjected to the proud man's contumely. We should be concerned to have it made a matter of notorious investigation whether the members of these colleges are positively wise and scientific, or relatively so only; whether their reputation must be supported by comparison with another branch of the profession, which, by their opposition, it will be inferred they labor to keep uneducated and ignorant; and whether their wealth and their influence must accumulate by an unavoidable consequence,—the accumulation of human misery.

To make the faculty acquainted with the proceedings in this great and even national question, was a promise formerly made to our readers. We have kept our word thus far, and shall from time to time pursue the same line. Of the probable termination of these proceedings, as to time, nothing determinate can be said; of their final success there is not, however, much doubt, because

1st, Experience has taught the Committee prudence and steady perseverance.

2d, Because the principle of the application to the legislature is founded on a PUBLIC GOOD.

Though the improvement of medical science be one of the most desirable of all things to society, that improvement becomes a dead letter and inefficient, if it is not generally understood and judiciously applied to practice; and unless the legislature provides, by proper laws, for the means of having it so understood and applied, industry vainly labors, and the light of genius becomes obscured by the surrounding gloom of ignorance. The reflection that in the present condition of medical police in this country, the advantages that arise from an improved state of the science are actually lost to society in a most extensive degree, might be expected to operate a concurrence of interests among the faculty, and to induce to the establishing of an efficient code of MEDICAL JURISPRUDENCE. That this concurrence does not at present exist is to be lamented, but it must not prevent our endeavor to promulgate such facts as come under our notice: it is a duty to make the best and the most of things as they are.

Since the publication of the preceding Report, in the month of January of this year, anatomy and physiology have received the additions of a work from Dr. Monro; an Essay on the Absorbents, by Mr. Pring; the Annual Oration at the Medical Society of London, by Mr. Saumarez, on the Principles of Physiological and Physical Science; and the first fasciculus of a work of considerable excellence on the Morbid Anatomy of the Liver, by Dr. Farre. The work of Dr. Monro, in three octavo volumes, and a volume of plates, is an abstract of the course of lectures on Anatomy and and Physiology, long delivered at the university of Edinburgh, by the celebrated individuals of that family. It embraces a very extended view of the subject, both as regards the natural state and healthy functions of the animal frame, and its diseased alterations and morbid actions. The little volume of Mr. Pring professes to comprise a history of the discovery of the absorbent system, a cursory view of the anatomy and physiology of that system of vessels and glands, an account of its morbid condition, and some inquiry into the relation that exists between the absorbing and secreting systems. The physiological and physical opinions of Mr. Saumarez have been so far explained in the preceding Numbers of our Journal, that few of our readers will remain quite unacquainted with them. In the essay now before us, Mr. Saumarez defines MAN to be "a rational soul in an animated body, which it employs as its instrument;" and this gives the occasion for exercising a severe castigation upon M. Richerand, who, in his Elements of Physiology, twice translated into English, asserts, "that our physical holds our moral nature under a strict and necessary dependence; that our vices and our virtues, sometimes produced, and often modified by education, are frequently too the result of organization." This assertion, which strikes to the root all moral restraint, and the assumed data on which it is founded, Mr. Saumarez attacks with the feeling and ardent eloquence of a man who is himself convinced of the existence of moral good and moral evil, and with the knowledge of an unprejudiced naturalist, who views a series of facts without being bound down by the confusion and bias of hypothesis. A principal dogma in the hypothesis of Richerand is, "*that the measure of the understanding is according to the number and perfection of the organs of sense.*" The French physiologist having thus far committed himself, it seemed not very difficult to refute his hypothesis by the simple fact as it really stands in nature. So far from Richerand's assumption being true, it is demonstrable that the *organs of sense are far more perfect in those animals that have the smallest than in those which have the greatest portion of understanding*. Mr. Saumarez produces a number of instances to prove this fact, and and we apprehend he is supported by general observation. As the friends of morality, of religion, and of social order, we become a party with this able advocate; and whatever may have been our opinion on some of his physical and physiological novelties, we believe him now placed on firm ground, and consider that we participate as auxiliaries in the credit of repelling the dangerous frivolities of a philosophy whose incessant object it is "*to elevate and to humanize the brute, whilst it degrades and brutalizes the man.*"

The first fasciculus, the only one which has yet appeared, of Dr. Farre's work on Morbid Alterations of Structure in the Liver, has considerably enriched this branch of the art, without, however, bringing with it the satisfaction of having suggested any remedial process: on the contrary, it shows that the two forms or species of disease of the liver, which this fasciculus describes under the terms *Tubera circumscripta*, and *Tubera diffusa*, are incurable. If we be considered as too fastidious when we object to the compound appellation *Morbid-Anatomy*, as designating with precision the investigation of diseases by anatomy, or as expressing unequivocally the anatomy of morbid parts, we are sure our readers will fully agree with us, as we do with the learned and ingenious author of this work, that inquiries thus prosecuted improve the diagnostic part of medicine, by connecting, as far as it can be done, the sign with the morbid change. It will not, however, be admitted, with Dr. Farre, that those two species of *Tubera* are absolutely incurable, but that investigations pursued with the candor, ingenuity, and science, manifested in this specimen of *Morbid-Anatomy*, (for we are still compelled to employ this equivocal term,) may eventually produce a degree of knowledge on this subject more favorable to the patient, and more honorable to the art.

Animal chemistry, closely connected with the preceding branch of natural science, has been much illustrated by Dr. Berzelius, professor of chemistry in the College of Medicine at Stockholm; and his labors have been rendered accessible to the English reader by a translation of his progress and present state of this art, by Dr. Brunnmark. In another quarter quarter Dr. Berzelius has also given to the English reader a general view of the composition of animal fluids. A paper, submitted to the Medical and Chirurgical Society of London, by this ingenious Swede, goes largely into the subject. Blood he makes the principal object of his inquiry. The fibrin is subjected to the action of caloric by boiling in water—to alcohol—to æther—to acetic acid—to weak muriatic, nitric, and sulphuric acid—and to caustic alkali. The chemical properties of the coloring matter are inquired into by a similar process; and a particular investigation is instituted for ascertaining the influence of iron for producing the color of this matter. If, as our countryman, Mr. Brande, has asserted, the coloring matter of blood is intirely independent of iron, this part of Dr. Berzelius' inquiry is extremely futile. The serum, the albumen, and the salts of the blood, become also subjects of this chemist's attention. If it is incompatible with our design to go into them with the ingenious author, it will not be unacceptable to our readers to be made acquainted with the results of Dr. Berzelius' labors on this part of his subject.

"Blood is composed of one portion, which is liquid and homogeneous, and of another which is only suspended, and spontaneously separates when at rest.

"The liquid part is a solution of much albumen and a little fibrin, both combined with soda. It also contains some other saline and animal substances, but in very small quantity.

"The portion which is suspended is the coloring matter. It differs from the albumen chiefly in its color, and its insolubility in serum. The color seems to be owing to *iron*, of which it contains $\frac{1}{3}$ per cent. of its weight, but which cannot be separated from it as long as it continues to be coloring matter. This separation can only be effected by combustion, or by the concentrated acids, both of which agents entirely decompose the substance with which the metal was combined. The coloring matter cannot be artificially produced by uniting albumen with red sub-phosphate of iron.

"Fibrin, albumen, and coloring matter, resemble each other so closely, that they may be considered as modifications of one and the same substance. These three substances produce, when decomposed, but do not contain, earthy phosphates and carbonate of lime; and indeed the entire blood contains in solution no earthy phosphate, except perhaps in too small a quantity to be detected.

"The albuminous contents of the blood will unite with acids, and produce compounds, that may be termed saline; these, when neutralized, will dissolve in water, but separate on adding an excess of water. Nitric acid, digested with the albuminous contents, forms an insoluble compound, consisting of albumen in an altered state, and of the nitric and malic acids. This property of combining with acids, is retained in some instances by the albumen, after it has undergone the changes produced in the secretory organs; as, for instance, in the peculiar matter of the bile, milk, &c.

"The blood contains no gelatine."

The secreted fluids, though obscure as to their mode of formation, are highly important in their results, either as performing some office in the animal economy, or as unloading it of something that would be oppressive if retained. These secreted fluids are divided into two classes, one of which is intended for some ulterior process in the system, the other to be directly discharged from the body. It is an extremely curious fact, that the fluids of the first class are all *alkalies*, and those of the second all *acids*.

The secreted fluids noticed by Dr. Berzelius, are bile, saliva, the mucus of the mucous membrane, mucus of the trachea, mucus of the gall-bladder, mucus of the urinary passage, fluids of serous membranes, humors of the eye, fluid of perspiration, urine, and milk. Why our author has neglected to notice one of the most important of the secreted fluids, *semen*, cannot easily be known.

Of the peculiar biliary matter, called *Pieromel* by Thouard, Berzelius gives this account. It has an excessively bitter taste, followed by some sweetness; the smell is also peculiar, and the color in most animals varies from green to greenish yellow. It is soluble in water, and its solubility is not in the least promoted by the alkali of bile; since, when this is neutralized by any acid, the peculiar matter does not separate; it also dissolves in alcohol in all proportions. Like the the albuminous materials of the blood of which this peculiar matter is composed, it will unite with acids, producing compounds of two degrees of saturation, and hence, of solubility. The acetous acid, which gives soluble compounds with the albumen of the blood, does the same with the peculiar matter of the bile; and hence this matter is not precipitated on adding this acid to bile, though it falls down on the addition of sulphuric, nitric, or muriatic acids. It is this sparingly soluble compound of biliary matter with a mineral acid which has been mistaken by many chemists for a resin; since it possesses the external characters of a resin, melts when heated, dissolves in spirit of wine, and is again precipitated by the addition of water. The alkalies, alkaline earths, and alkaline acetates, decompose and dissolve it: the former, by depriving it of its combined acid; the latter, by furnishing it with acetous acid, which renders it soluble in water.

The peculiar matter of the bile also combines with many metallic oxides into a pulverulent mass; and the resiniform compound of this matter, and any of the mineral acids, often forms with the same oxides a substance like a plaster, resembling in this respect also the true resins.

The biliary matter may be obtained pure by mixing fresh bile with sulphuric acid diluted with three or four times its weight of water. A yellow precipitate first appears, which must be allowed to subside and be removed; then continue to add fresh acid as long as any precipitate is formed; heat the mixture gently for some hours, and afterward decant the fluid part, and thoroughly edulcorate the green resin which is left. This resin reddens litmus, and is partially and sparingly soluble in water. It may be deprived of its acid in two ways: one of them is by digesting it with carbonate of barytes in water, whereby the carbonate is decomposed, and the water forms a green solution, possessing all the peculiar characters of bile: the other way is by dissolving it in alcohol, and digesting the solution, either with carbonate of potass or carbonate of lime, till it no longer reddens litmus, and then evaporating it to dryness. Either of these methods will give pure biliary matter.

This peculiar biliary matter, when pure, resembles exactly desiccated bile. Being soluble in alcohol it might be supposed that it would dissolve in æther, but æther only changes it to a very fetid adipocirous substance, exactly as it acts upon the albuminous matter of the blood. It is surprising that biliary matter gives no ammonia by distractive distillation, therefore it contains no azote; no vestige of azote in any other of the constituent parts of the bile, nor does bile contain ammonia.

Berzelius gives the following result of his analysis of the bile:

| | |
|---|---|
| Water | 907·4 |
| Biliary matter | 80·0 |
| Mucus of the gall-bladder, dissolved in the bile | 3·0 |
| Alkalies and salts, common to all secreted fluids | 9·6 |
| | 1000·0 |

The researches which have been directed to animal substances by the chemists, it will readily be conceived, would not leave untouched that part of the frame which has been deemed the laboratory and store-house of intellect. The peculiar texture, appearance, and believed functions of the brain, have accordingly excited inquiries, ingenious and elaborate, which, we fear, have not yet terminated in any positive and indisputable conclusion. Among the various methods taken to examine the structure, and ascertain, by that means, the functions and actions of the brain and nerves, chemical analysis has been employed, with what effect future observations will determine. The abilities of M. Vauquelin in this department of science, are too well known to suffer his *Analysis of the Cerebral Matter of Man and other Animals*, to remain unnoticed. His predecessors, Gurman, Burrhus, Thouret, Fourcroy, &c. in the same inquiry, have not anticipated his labors. Elaborate minuteness, rigid accuracy, and an accustomed eye, combine to render this analysis important, as ascertaining the constituents of the cerebral organ; but they do not develope its physiology.*

* This analysis of the brain, by M. Vauquelin, we think of sufficient importance to demand the insertion of the Memoir, which will be found in another part of our Journal.

The

The connection of agriculture with, and its influence on the health of man and other animals, both as it regards alterations produced by its processes on the earth's surface, and by its improvement of alimentary substances, necessarily makes it interesting to the professors of medical science; and as a branch of chemistry it approximates still more to the healing art. The deserved reputation of Sir H. Davy gives to every production of his pen great value and extensive currency: and it is with peculiar satisfaction we observe his talent for investigating nature, applied to the chemistry of agriculture. In his "*Elements of Agricultural Chemistry*," recently published, a very extensive experimental and scientific view is taken of this important subject. An examination of the general powers of matter upon vegetation, whether gravitation, cohesion, chemical attraction, heat, light, or electricity, is instituted; the organization of plants, the constituent parts of soils, and the nature and constitution of the atmosphere, with its influence on vegetable life, are investigated with experienced acumen. An extensive experimental inquiry is made into the product and nutritive qualities of different grasses, and other plants used as the food of animals. With this cursory notice of one of the most valuable publications of the preceding six months, it does not seem to be improper to connect an elegant Essay on the Philosophy, Study, and Use, of Natural History, noticed in our preceding Number. The objects and views of this interesting little volume are most important. The study of natural history affords a sober and certain satisfaction far beyond any other human pursuit. To follow effects up to their causes, and from thence to develope other effects; to collect, arrange, and generalize individual facts, and upon them to found or explain principles, seems to be the proper employment of human intellect. "To trace," as this author happily expresses, "the footsteps of God, the eternal, the infinite, the omniscient, and omnipotent, throughout all Nature—to be initiated in mysteries and laws which produce effects that are necessary for our welfare, or that conduce to our happiness—to seek the knowledge of *His* designs, and of *His* works, to the end that we might more properly properly appreciate the various objects, both animated and inanimated, by which we are surrounded; and to regulate our duties and our pleasures by that knowledge," is a direction and occupation of the mind devoutly to be wished. Among much valuable observation contained in this Essay, we notice a constant effort to direct the naturalist's views beyond the surface of nature; to show him that external lineaments are the least important of the objects subjected to his inquiries; and that his mind should be directed to explain the connections, dependencies, capacities, and final destinations, of the myriads of forms with which the CREATOR has peopled this earth. "It should be the business of the true naturalist," he says, "to study the works of GOD with a view, and an earnest desire to understand *His* designs respecting them;—that he should endeavor to learn their utility, both general and particular, rather than to make himself acquainted with their mere external character, their names, or the classes into which they appear naturally separated, though linked;—that he should regard all created beings as one vast family united together for some great end; over whom, as lord of the whole, he should extend the offices of benevolence, rather than the spirit of persecution;—that in using, applying, or changing natural bodies for his own purposes, he should be careful to fulfil, rather than to counteract, the will of the GREAT FATHER and ARCHITECT of the UNIVERSE; as far, at least, as can be felt and discovered: and, above all, that he should seek to know his own duties in the midst of that beautiful creation, which, like a celestial garden, has been spread out for his footsteps, and given to him as an inheritance."

The practical part of medicine and surgery have each, within the past six months, received some interesting if not important additions.

The destructive effects of that form of fever believed to arise from some certain, or rather uncertain, state of the atmosphere over marshy lands, as its cause, is of so much importance, especially in military expeditions, that every inquiry which tends to clear away the obscurity yet hanging upon upon the subject, claims particular attention. In the expedition to the island of Walcheren in 1809, the effects of this destructive material, whatever it may be, were felt to a most alarming extent. Many of the physicians and surgeons attached to that army have given full details of the morbid effects produced by this material, but none have yet been able to detect any properties peculiar to it beyond its power of occasioning remittent and intermittent fever. Of its constituent principles we yet remain ignorant; but it seems unquestionable that something is emitted by or exhaled from certain marshy ground capable of producing, most extensively, fevers of a violent and fatal nature. Though Sir Gilbert Blane has not removed this difficulty, we are indebted to him for concentrating many facts relative to the operation of this poison, and on the methods employed to obviate its deleterious effects. Our limits will only permit us to attend to the natural history of marsh miasmata. On the facts stated by Sir Gilbert Blane, (Medico-Chir. Trans. vol. iii.) it appears that the whole island of Walcheren, with the exception of some mounds of sand on the western shore, is a dead flat, below the level of the sea at high water, and preserved from inundation by artificial embankments. The soil consists of a fine white sand, known in the eastern counties of England by the name of *silt*,* and about a third part of clay. It is divided into small square inclosures by ditches, which

---

* *Silt is not a fine white sand*, but a deposition from sea-water of a brownish yellow in the English counties Lincoln, Cambridge, and Norfolk, where it is found in extensive fenny tracts lying toward the sea. In the fens of these counties the alluviæ are of two very distinct kinds. The alluvial deposition in that part which lies nearest to the highlands, and which extends several miles into the great level, is entirely, or very generally, a black substance, provincially termed Moor: it lies from one to four feet thick upon a sub-stratum of clay commonly, but sometimes of gravel, and is cut into fuel under the denomination of Turf. The alluvial deposition in that part lying toward the sea, is a soft yellow-brown sand denominated Silt: it is extremely fertile, and constitutes the most valuable pasturage of the whole district. Remittent and intermittent fevers are extremely frequent which serve as drains; and these were, in the endemic season, about two thirds full of turbid water. They emitted no smell, but a disagreeable effluvia arose from stagnating pools. The soil seems a mass of alluvial matter like the deltas of great rivers; and the whole islands of Zealand were, probably, formed by the *detritus* carried down by the Rhine and the Scheldt. The poisonous exhalations from soils thus formed, is not animal putrefaction, for it is ascertained that those who are exposed to putrid vapors, such as anatomists and tanners, are not affected with complaints like those arising from marsh miasmata. Water in a state of stagnation, without any ascertainable principle of contamination, seems to generate these poisonous exhalations. The delta of the Nile does not generate intermittent fever, because the inundations of that river obviate the effects of stagnation; while, in the island of Minorca, in places consisting of a very thin soil on a rocky bottom, this fever appears in its severest form, in consequence of stagnant water in channels and pools. The miasmata in Zealand are more noxious than the like exhalations in England. The exhalations of the soil in tropical climates are still more malignant than those of Zealand. In the West Indies, and at Calcutta, ships at the distance of three thousand feet from swampy shores were affected by the noxious exhalations. Many facts are stated by Sir Gilbert Blane to show the extent to which the deleterious miasmata may produce its effect, and the direction which it is most disposed to take; and others, still more valuable, go to prove that certain artificial changes may deprive particular localities of the property by which they generate this poison. The principle of this melioration is doubtless an improved drainage. There are still some facts opposed to this principle, which must be cleared away before it is incontrovertibly established.

---

frequent on both these surfaces; but they occur most in the driest state of the *fens*. In hot and dry autumns the endemic most prevails, and when the surface of the great level, from the texture of its soil, through which water percolates with rapidity, is extremely arid.

Some particular facts of considerable importance in the treatment of individual diseases have fallen under our notice. Perhaps the most valuable of these are related by Dr. Sutton, who has established assertions by the evidence of numerous cases. The use of opium in *Delirium tremens* he appears to have ascertained in a very decisive manner, and to have suggested an improved treatment of peritoneal inflammation, deserving serious consideration.

The disease denominated *Delirium tremens* by Dr. Sutton, is described as often coming on slowly. For some days previous to the distinct commencement of the malady, the patient complains of being unwell, with loathing of food, listlessness, debility, and want of comfortable rest. He has pain in the head, and sometimes vomits, and is dejected. The pulse, in the commencement of the disease, commonly is not quick; but may be frequently observed to have an unsteady fluttering. There is not much heat on the skin, and the tongue is generally furred and moist. In this stage of the disease, the patient has very little disposition to lie down for any length of time, but is ever uneasy, and desirous of a change of position; and there is a general agitation of the frame, with tremors of the hands. Associated with these is a wavering of the mind; and, if the disease proceeds, this becomes every day more manifest. As the disease advances, the faculties do not, generally, show themselves in disorder by any extravagance of thought, but by fatiguing conversations on common affairs frequently repeated; and by broken discourses, caused evidently by confusion of intellect. In the further progress of the disease, the patient discovers great anxiety of mind about his affairs, appears ever desirous to be where business is, and makes great, repeated, and violent efforts to liberate himself from those about him, if under restraint, in order to accomplish the objects that press most forcibly on his mind. These exertions are, however, not made in opposition to others, though violent, with either malignity or ill-nature; nor does the patient mark his restraints with the appearance of much anger or displeasure. He seems to be forgetful of what has immediately passed, and only to be propelled to action by those strong impressions on his mind respecting the objects just stated. In other respects he is tractable, and there is seldom any difficulty in administering medicine to him. In this situation he loses the sensation of pain; and when in a considerable degree of this delirium, knows momentarily those about him of his family and friends. *The tremors of the hands, which constantly accompany this complaint, are now great, with unceasing workings and elevations of the tendons at the wrists; to which are frequently associated subsultus tendinum, and often singultus.* By the action of the tendons at the wrist, the hands are drawn inwards, sometimes to such extent, joined to the constant tremors, as to allow a very imperfect knowledge of the pulse. When the patient is at all still, he is constantly picking the bed clothes, and in various motions with his hands. The evacuations are unconsciously rejected, in the height of the paroxysm. The pulse at this time becomes very rapid, and may appear to be more debilitated than it really is, from the difficulty of ascertaining its actual state. Accompanying the exertions at this time made, there is generally a most profuse sweat, which is commonly clammy and cold; with sometimes an offensive odor. The heat of the skin varies much, but is seldom intense; and the tongue is not often dry, or the patient thirsty. The general appearance of the countenance is dull, and the eye frequently suffused. The state of the bowels vary; but, during the violence of the disease, frequent stools are not common. In the height of the paroxysm, the patient is in an unremitting state of watchfulness, which continues until the disease is alleviated, or is succeeded by insensibility, which may partake of coma or apoplexy, ending in death.

This disease will continue, with great violence, from three days to a week, and with moderate symptoms for a longer time, and is sometimes seen in the form of a chronic affection.

The curative intention is to procure quiet and sleep; these being effected, the disease subsides. Opium, in large doses, two grains of the extract every two hours, and in some some instances every hour, was given with the happiest consequences.

In peritoneal inflammation, instead of the hot bath, hot fomentations, and the application of heat by various methods, Dr. Sutton employs COLD; and he relates many cases in which the abstraction of heat by the application of a cold lotion* to the abdominal parietes, was followed by the most decisive benefit.

We are disposed to consider these as instances of practical improvements, since the publication of our former Report, of great importance. The *modus operandi* of opium in *Delirium tremens*, Dr. Sutton does not explain; but the beneficial application of cold in *Peritonitis*, seems to have an obvious solution. In all topical inflammation there is an increase of temperature in the part, invariably occurring as a *sine qua non* of that morbid state. It is a self-evident proposition, that, by the abstraction of caloric, this increased temperature will be reduced, and one essential part of the diseased action, probably its most essential, removed.

In the employment of *caloric* and *frigoric* as articles of the materia medica, we have yet much to learn. As productive of diseased actions, these principles, substances, or qualities, are but little understood: and our conclusions respecting their *modus operandi*, in existing morbid effects, are often extremely illogical.

The expectations, formed on facts stated to have occurred in India, of the possibility of curing hydrophobia by excessive bleeding, are still kept alive. Since these facts have been given to the public, Dr. O'Donnel, of Uxbridge, has printed cases of this dreadful malady, which, like all others, we fear, of the specific disease, terminated fatally. This gentleman favors the opinion that copious and even excessive abstraction of blood affords the most probable means of resisting the effects of this formidable poison. One circumstance connected with the case of Joseph Watson, related by Dr. O'Donnel, (Med. and Phys. Jour. vol. xxix. p. 491) is entitled still fur-

---

* The lotion employed by Dr. Sutton was made with Mist. Camph. ℥xii. Liq. Ammon. Acet. ℥iij. Spir. Vin. Ten. ℥j.

ther to arrest attention, not only as an interesting fact in the history of this disease, but as tending to excite an essential precaution in the diseases which occur to dogs. The bitch by which Joseph Watson was bitten, never had the symptoms of *Rabies*, according to the apprehension of those who saw her; and yet her bite was followed by that disease in all the animals on which it was inflicted, showing very distinctly, that the virus may exist in an individual in all its virulent activity, without manifesting its characteristic features on that individual.

The *Memoires de Chirurgie Militaire* of M. Larrey, from the large scale of observation presented to their author in his various campaigns with the French armies, make a valuable addition to the chirurgical literature of this period. Almost every variety of disease and accident occurring to the soldier on actual service, receives illustration from the collections of this accomplished surgeon. The typhus of camps and hospitals, the effects of fatigue and privation, the consequences of exposure to great changes of temperature, are explained in the *Chirurgie Militaire* with minuteness and precision. The losses sustained in the French armies by the coldness of the Russian climate in the latter part of 1812, though upon a scale to excite and interest curiosity, had been before felt in the campaign in Poland. "Few soldiers of the advanced guard, after the battle of Eylau, escaped the effects of severe cold upon their toes, feet, nose, or eyes." There is a remarkable fact connected with this, and which will go far toward fixing the floating opinions at present existing, on the effects produced by heat after cold. Not a single man suffered from the frost during the time the mercury was at 20° and downwards to 0° of Fahrenheit; but as soon as the mercury rose above 32°, and a thaw began, many of the soldiers complained of violent pain and numbness in their extremities, and the parts assumed a deep red color. Those who exposed themselves to the fire fared the worst; those who rubbed the frost-bitten parts with snow, and brandy and water, escaped mortification. The opinion of *M. Larrey* that *cold* is only the predisposing cause of mortification, and that the sudden application of a high temperature perature to the torpid parts, is essential to the production of inflammation and gangrene, is most likely true. Our readers will perceive that this extreme case covers a multitude of minor instances, where neither the cold nor the heat have been extreme, but where still disease has been produced.

The method employed by Mr. Adams, for the cure of Egyptian ophthalmia, and related at p. 302 of the preceding volume of this Journal, deserves the attention of the faculty, as being successful in arresting the progress of a disease so rapidly destructive of the organ of vision.

An instance of the detection of an extraordinary imposition, which took place in April last, deserves to be noticed in the record of passing events. *Ann Moore*, a woman residing at Tutbury in Staffordshire, assumed the property of living without food for the five or six past years. This she managed with such dexterity, that many worthy persons were so far imposed on as to place implicit confidence in her. In 1808 this woman submitted to an investigation of her case by a watch set over her for several days. The result of this was so favorable, that her assertion of absolute abstinence found general credence. In this state of public opinion, Dr. Henderson, of London, saw her in the autumn of 1812. The observations he made led him to the conclusion of her being an impostor; and his remarks, first printed in this Journal, and afterward in a separate pamphlet, were so pointed, as to compel her again to submit to be watched. The result was a full confession of her guilt. It does appear, however, that this woman, partly perhaps from habit, and partly from some altered state of the functions of the stomach, did possess the power of abstaining from food, both solid and fluid, for many days together; and in her last effort to continue her imposition, it is ascertained that she remained nine days and nights without any sort of nutriment. The annals of the medical art afford many instances of similar impositions; but few have had more publicity, been more generally believed, or more fully detected, than this of *Ann Moore*.

On the subject of medical science generally, a valuable volume by Dr. Thomas Young, under the title of " An Introduction troduction to Medical Literature," has appeared since last January. This assumes, in a degree, the form of a Bibliotheca; contains numerous references to authors, under particular classes; but is principally occupied in supplying a more correct Nosology, founded on the principles of the *Philosophia Botanica* of Linnæus. The defects in Cullen's Nosology induced Dr. Young to undertake this laborious and difficult office; how far he has succeeded must be left to the profession to determine: but that he has brought together a highly useful body of reference, and has also enriched his work with several ingenious essays, will hardly be controverted. To form the accomplished professor and the able practitioner in a science so important, comprehensive, abstruse, and intricate, as that of medicine, requires not an attentive observation of *Nature* alone, but a full view of whatever has been discovered, invented, or illustrated, by preceding ages. With such difficulties in the path to medical knowledge, the guide presented to the English student by Dr. Young, deserves to be held in high estimation. It is not, however, by such aids, singly, that medical science can be brought to perfection; an union of minds, an accordance of exertions, a participation of powers, and a dismissal of disgraceful jealousies, are essential to the consummation of this great object.

*June* 1, 1813.

